# Early citation dynamics and predictors of time to first citation in Ecuadorian medical publications: a survival analysis of research visibility

**DOI:** 10.3389/frma.2026.1816766

**Published:** 2026-06-29

**Authors:** Paloma González, Marco Coral, Martha Fors

**Affiliations:** 1 Biblioteca, Universidad de Las Américas, Quito, Ecuador; 2 Facultad de Medicina Veterinaria y Agronomía, Universidad UTE, Quito, Ecuador; 3 One Health Research Group, Facultad de Ciencias de la Salud, Universidad de Las Américas, Quito, Ecuador

**Keywords:** Ecuador, medical research, scientometrics, survival analysis, time to first citation

## Abstract

**Background:**

Citation-based indicators are widely used to assess scientific impact, yet they often overlook the temporal dynamics of knowledge dissemination. Time to first citation has gained relevance as an indicator of early research visibility, particularly in emerging research systems.

**Objective:**

To analyze time to first citation of Ecuadorian medical publications and identify publication characteristics associated with citation timing using a survival analysis approach.

**Methods:**

A scientometric study was conducted on Ecuadorian medical publications indexed between 2015 and 2024, with citation follow-up through December 31, 2024. Descriptive statistics summarized publication characteristics and citation counts at 2 and 5 years. Time to first citation was analyzed using Kaplan–Meier estimators and Cox proportional hazards regression models, accounting for right-censored data.

**Results:**

A total of 3,968 publications were included. Most articles were published in open access journals (63.0%) and involved international collaboration (69.3%). Publications were predominantly in English (85.1%). Most records were original research articles (2,917; 73.5%), while 597 (15.0%) were review articles; article type information was unavailable for 454 records (11.4%). The median number of cumulative citations was 3 (IQR 1–7) at 2 years and 6 (IQR 3–14) at 5 years. Kaplan–Meier analysis showed that approximately 50% of articles received their first citation within the first year, and most were cited within 4 years. In the multivariable Cox regression analysis, international collaboration (*HR* = 0.81; 95% *CI*: 0.67–0.97), non-English language (*HR* = 0.53; 95% *CI*: 0.42–0.65), original article type (*HR* = 1.40; 95% *CI*: 1.09–1.79), and number of authors (*HR* = 1.001; 95% *CI*: 1.001–1.002) were significantly associated with time to first citation, whereas open access status was not independently associated with citation timing after adjustment.

**Conclusions:**

Ecuadorian medical publications demonstrated relatively rapid early citation uptake. Publication language, article type, international collaboration, and authorship characteristics were associated with time to first citation. These findings should be interpreted cautiously given the observational design and limitations in metadata completeness. The study highlights structural factors that may influence early research visibility in emerging research systems.

## 1 Introduction

Understanding how quickly scientific publications receive their first citations is important for assessing early research visibility and scholarly uptake. Although citation counts are widely used as indicators of scientific influence, they reflect not only intellectual impact but also social factors and disciplinary conventions (Bornmann and Daniel, 2008). For this reason, temporal indicators such as time to first citation provide a valuable complement to traditional citation metrics.

Time-based approaches to citation analysis have gained increasing attention as measures of how rapid knowledge diffuses within academic communities. Survival-analysis methods, including Kaplan–Meier estimators and Cox proportional hazards models, are well suited for modeling time to first citation while accounting for right-censoring, which occurs when a publication has not yet received its first citation by the end of follow-up. Recent studies support the use of these methods across disciplines. Giordano et al. (2025) applied survival analysis to scientific journals and identified distinct temporal citation patterns, while Srisawad et al. (2025) showed in medical research that journal and authorship characteristics significantly predicted earlier citation.

Structural dissemination factors also influence early citation. Open-access articles tend to receive more citations and more rapidly than subscription-based publications. A systematic review by Langham-Putrow et al. (2021) confirmed a significant citation advantage for open access. More recent studies reported similar findings in medicine and dentistry (Yi et al., 2024; Yu et al., 2022). Likewise, collaboration networks, particularly international co-authorship can enhance visibility and citation likelihood. In South America, De Lima et al. (2025) found that international collaboration improved citation metrics in palliative care research.

Despite growing interest in citation dynamics, evidence from low- and middle-income countries (LMICs), especially in Latin America, remains limited. Although regional scientific productivity has increased, Latin America is still underrepresented in scientometric evaluations. Maldonado et al. (2025) highlighted both rising emergency medicine research output and persistent structural barriers affecting visibility in the region.

Ecuador represents an important but understudied setting. Although biomedical publication output has grown in recent years, particularly after the COVID-19 pandemic, no previous study has systematically examined how quickly Ecuadorian medical publications receive their first citations.

This study addresses that gap by applying survival-analysis methods to medical publications with Ecuadorian institutional affiliations. Specifically, we aimed to (1) estimate time to first citation, (2) identify publication and collaboration characteristics associated with shorter citation delays, and (3) describe cumulative citation counts at 2 and 5 years after publication.

## 2 Methodology

### 2.1 Study design

A retrospective bibliometric cohort study was conducted to evaluate the time to first citation of Ecuadorian medical publications using time-to-event analytical methods. Survival analysis was selected because it enables estimation of citation delay while appropriately accounting for right-censored observations, defined as publications that had not received a first citation by the end of the follow-up period.

### 2.2 Data source and eligibility criteria

Data were retrieved from the Clarivate Web of Science Core Collection, selected for its international coverage, standardized indexing, and citation-tracking functionality. Eligible records were original articles and review articles published between January 1, 2015, and December 31, 2024, with at least one author affiliated with an institution in Ecuador. The study period (2015–2024) was selected to ensure data consistency and comparability over time. Publications prior to 2015 were excluded due to greater variability in indexing practices, metadata completeness, and journal coverage in earlier records within the Web of Science Core Collection (WoSCC), particularly for emerging research systems such as Ecuador. The study was restricted to publications classified within the subject area of Medicine to preserve disciplinary comparability, as citation practices differ substantially across scientific fields (Ioannidis et al., 2016). Only records published in English, Spanish, French, or Portuguese were included because these languages represented the overwhelming majority of eligible publications and allowed reliable classification of bibliographic metadata. Additionally, language was categorized as “English” vs. “Non-English” for regression analyses. Editorials, letters, meeting abstracts, proceedings papers, notes, and other non-citable document types were excluded. The final search strategy applied in the advanced search interface was based on document type, country affiliation, publication year, language, and subject category filters.

### 2.3 Variables and data extraction/ outcome definition and follow-up

For each eligible publication, the following variables were extracted: publication year, year of first citation, total citation count, number of authors, document type (article or review), publication language, open access status and presence of international collaboration The year of first citation was obtained automatically through the Citation Report function of the Web of Science Core Collection (WoSCC), which provides annual citation histories for indexed records rather than exact citation dates. International collaboration was defined as the presence of at least one co-author affiliated with an institution outside Ecuador. Author affiliations and country information were obtained from WoSCC metadata, and publications were manually classified as involving international collaboration (“yes”/”no”) based on these affiliations. Journal quartile information was not available in the exported Web of Science Core Collection metadata. Access to Journal Citation Reports (JCR) quartile data for the complete dataset was not available, and manual retrieval of quartile classifications for all included publications was not feasible due to the large number of records. To enhance data completeness, a manual verification process was conducted for records with an available Digital Object Identifier (DOI). For these articles, the original publication sources (e.g., journal websites or publisher platforms) were reviewed to retrieve missing information related to Open Access status, article type, language, and collaboration characteristics when such data were not fully indexed in the Web of Science Core Collection (WoSCC). Despite this additional step, some variables remained unavailable due to limitations in database indexing or inconsistencies in reporting across sources. These cases were categorized as “Not available” in the final dataset. Although the search strategy was restricted to original articles and review articles, document type information was not consistently available in the exported metadata for all records. When article type could not be verified through manual review, publications were classified as “Not available” for descriptive analyses.

The primary outcome was time to first citation, measured in years from the publication year to the year in which the first citation was recorded. Because citation data in WoSCC were available only by calendar year, publications receiving their first citation within the same year (By December 31) as publication were assigned a time-to-first-citation value of zero years. Consequently, the analysis reflects annual rather than exact temporal resolution (treated as right-censored at the end of follow-up.). The publication window (2015–2024) was selected to ensure a contemporary cohort while maximizing available follow-up time for citation assessment through December 31, 2024.

### 2.4 Data management

Extracted records were cleaned, harmonized, and checked for duplicates and inconsistencies prior to analysis. Variables with unavailable metadata were retained as missing categories for descriptive analyses. For multivariable regression models, complete-case analysis was performed. To improve data completeness, records with a Digital Object Identifier (DOI) underwent manual verification using original publication sources to retrieve missing information on Open Access status, article type, language and international collaboration. International collaboration was manually completed when possible. Remaining missing values were categorized as “Not available.”

### 2.5 Statistical analysis

Descriptive statistics were used to summarize publication characteristics. Categorical variables were reported as frequencies and percentages, whereas continuous variables were summarized using medians and interquartile ranges (IQRs), given the non-normal distribution of citation counts. Citation accumulation was additionally summarized at 2 and 5-year intervals. The probability of remaining uncited over time was estimated using Kaplan–Meier methods. Median time to first citation and corresponding 95% confidence intervals (95% *CI*) were calculated where estimable. Univariable Cox regression analyses were initially performed for each covariate independently. Subsequently, a multivariable Cox regression model was constructed including all variables considered theoretically relevant and available in the dataset. This approach was used to estimate adjusted hazard ratios (HRs) and corresponding 95% confidence intervals (95% CIs), while controlling for the potential confounding effects of the other publication characteristics included in the analysis. Covariates included number of authors, international collaboration, article type, open access status, and language. The proportional hazards assumption was assessed using Schoenfeld residuals and graphical inspection of log-minus-log survival curves. All statistical tests were two-sided, and statistical significance was defined as *p* < 0.05. Analyses were conducted using R version 4.4.1 and SPSS version 26. Missing metadata were treated as separate categories for descriptive analyses; complete-case models were used in regression analyses.

## 3 Results

Table 1 summarizes the descriptive characteristics of the 3,968 Ecuadorian medical publications included in the study. Most articles were published under an open access model (2,499; 63.0%) and involved international collaboration (2,750; 69.3%). English was the predominant language of publication (3,378; 85.1%), while non-English publications accounted for 590 records (14.9%). Most records were original research articles (2,917; 73.5%), while 597 (15.0%) were review articles; article type information was unavailable for 454 records (11.4%). The median number of authors was 7 (IQR 5–11). Regarding citation impact, the median cumulative citation count was 3 (IQR 1–7) at 2 years after publication and 6 (IQR 3–14) at 5 years (Table 1).

**Table 1:** Descriptive characteristics of the analyzed publications.

Variable	Category	*n* (%)
Open access	Yes	2,499 (63.0)
	No	1,091 (27.5)
	Not available	378 (9.5)
International collaboration	Yes	2,750 (69.3)
	No	594 (15.0)
	Not available	624 (15.7)
Language	English	3,378 (85.1)
	Non-English	590 (14.9)
Article type	Original article	2,917 (73.5)
	Review	597 (15.0)
	Not available	454 (11.4)
Citations	Cumulative citations at 2 years	Median 3 (IQR 1–7)
	Cumulative citations at 5 years	Median 6 (IQR 3–14)

**Figure 1:**
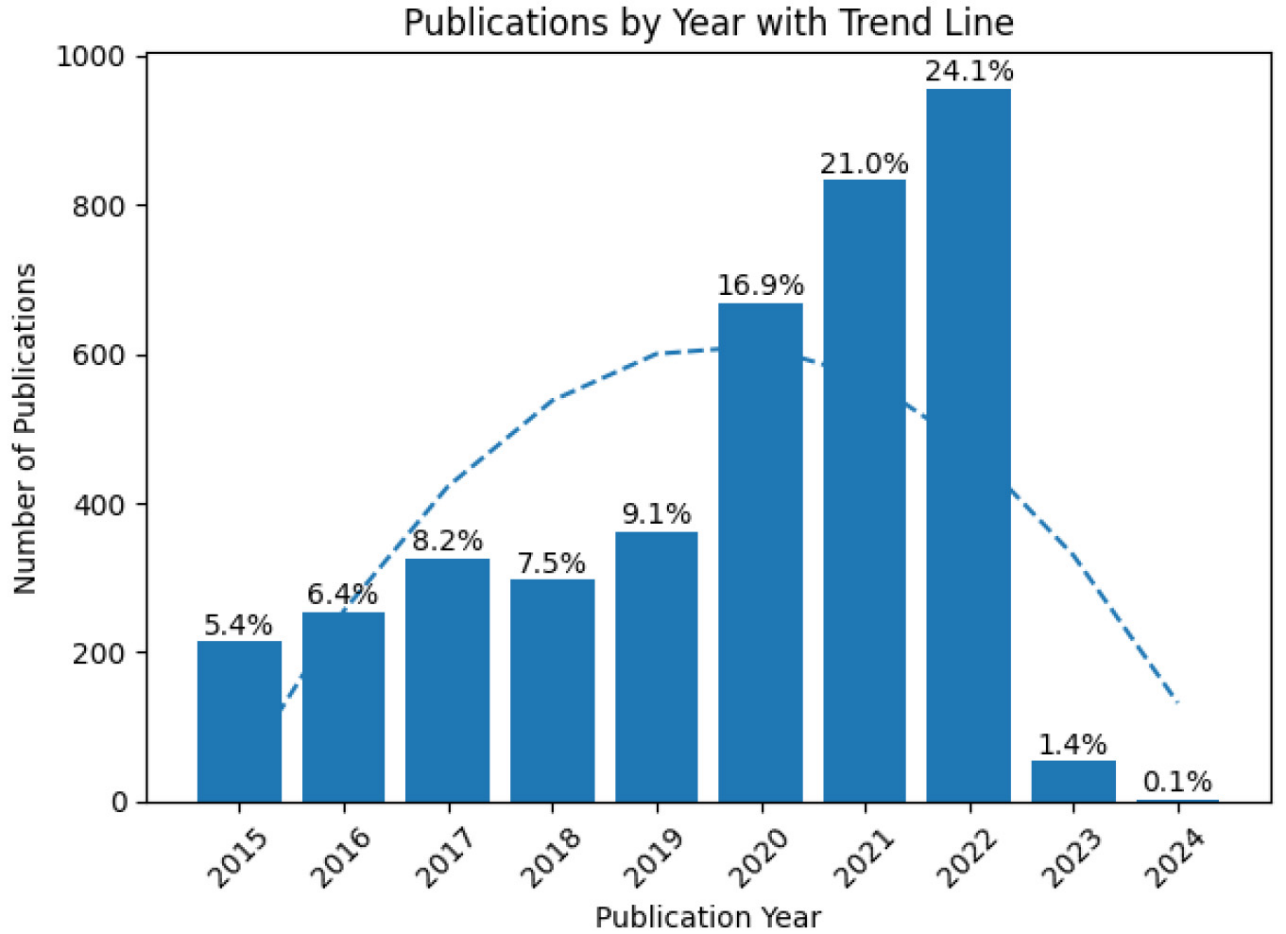
Trends in annual publication output, 2015–2024. Annual distribution of publications from 2015 to 2024, with a polynomial trend line. Research output increased steadily from 2015 to 2019, followed by a sharp rise beginning in 2020 that peaked in 2022. The decline in 2024 reflects incomplete data collection rather than a true reduction in productivity.

Figure 1 illustrates the annual distribution of Ecuadorian medical publications included in the study between 2015 and 2024. Publication output increased gradually from 214 articles in 2015 (5.4%) to 362 articles in 2019 (9.1%). A marked rise was observed beginning in 2020, with 669 publications (16.9%), followed by further increases in 2021 (833; 21.0%) and a peak in 2022 (956; 24.1%). In contrast, the number of publications decreased sharply in 2023 (54; 1.4%) and 2024 (4; 0.1%). The dashed trend line highlights the overall growth in publication output up to 2022, followed by a decline in the final years of the observation period.

**Figure 2:**
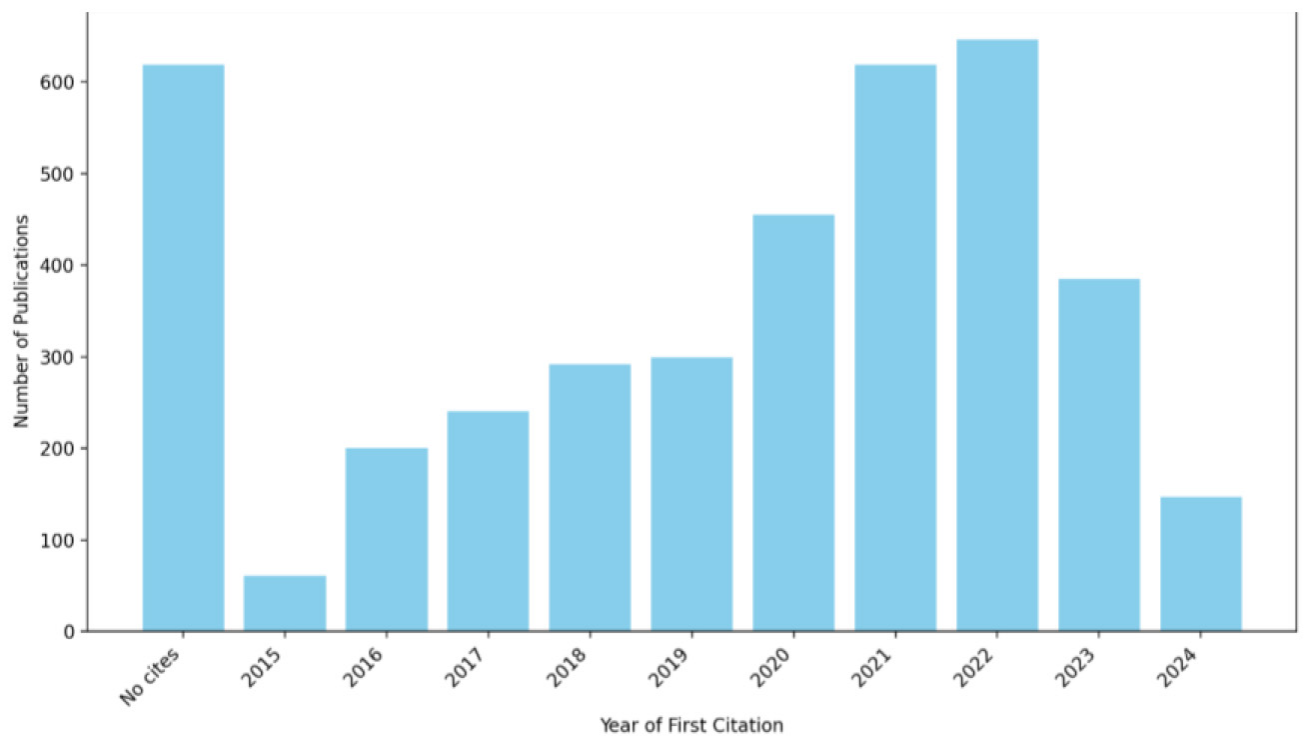
Distribution of first citations by year (2015–2024). This bar chart shows the number of publications according to the year in which they received their first citation. The category “No cites” represents articles that had not received any citations by the end of the observation period. The distribution indicates a progressive increase in first-time citations from 2015 onward, with a marked rise between 2020 and 2022. The lower counts observed for 2023 and 2024 are likely to reflect citation delay and incomplete follow-up rather than reduced research impact.

Figure 2 shows the distribution of first citations by year from 2015 to 2024, including a “No cites” category. The highest proportions of first citations were observed in 2022 (646; 16.3%), followed by 2021 (619; 15.6%) and 2020 (455; 11.5%). First citations increased notably from 2018 onward, peaking during 2020–2022. Lower counts were observed in 2023 and 2024 compared with previous years. The “No cites” category represented 15.6% of records, corresponding to publications that had not yet received a first citation by the end of follow-up (Figure 2).

**Figure 3:**
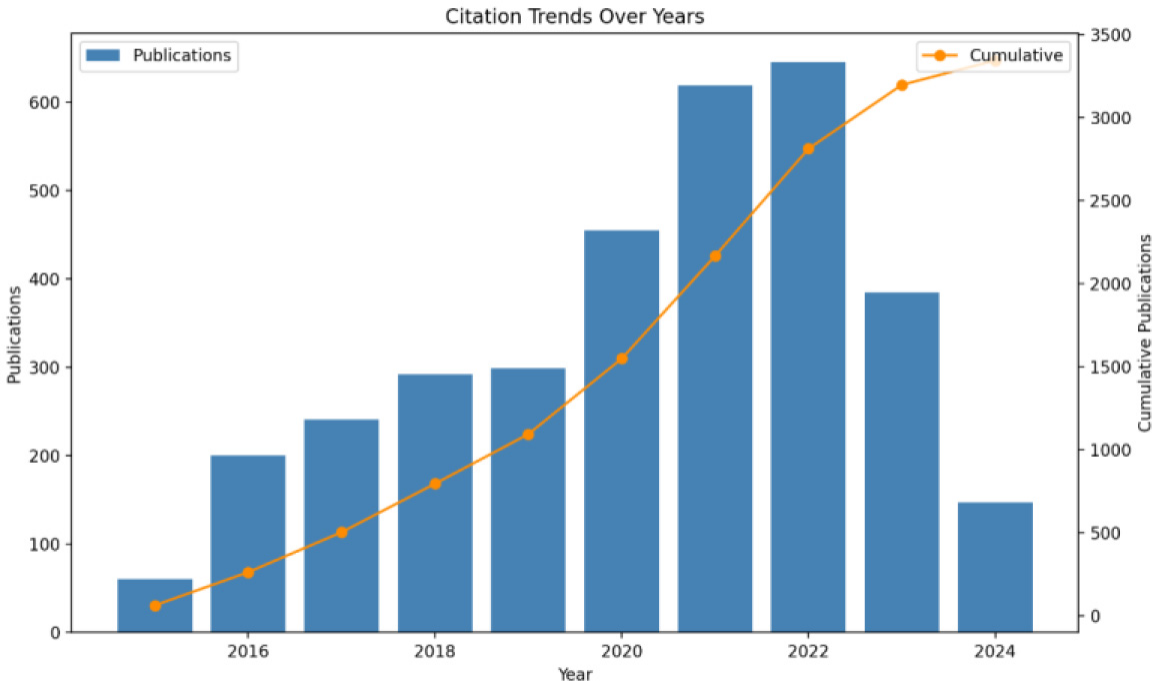
Trends in first-time citation activity and cumulative growth of cited publications (2015–2024). The bars represent the number of publications that received their first citation each year (left axis). The orange line shows the cumulative total of all publications cited for the first time up to that year (right axis), highlighting the overall growth in the body of cited literature. Notice the steady growth from 2015 to 2019, a surge between 2020 and 2022, and a slowdown in new citations during 2023–2024.

Figure 3 illustrates annual first citations and their cumulative growth over time. The bars (left axis) represent the number of publications receiving their first citation in each calendar year, while the orange line (right axis) shows the cumulative total of newly cited publications. Between 2015 and 2019, growth was steady but modest, with approximately 40–60 publications gaining a first citation annually. A marked increase was observed from 2020 to 2022, when yearly counts nearly doubled and the cumulative curve rose sharply. In contrast, lower counts in 2023 and 2024 resulted in a flattening of the cumulative trend, (Figure 3).

**Figure 4:**
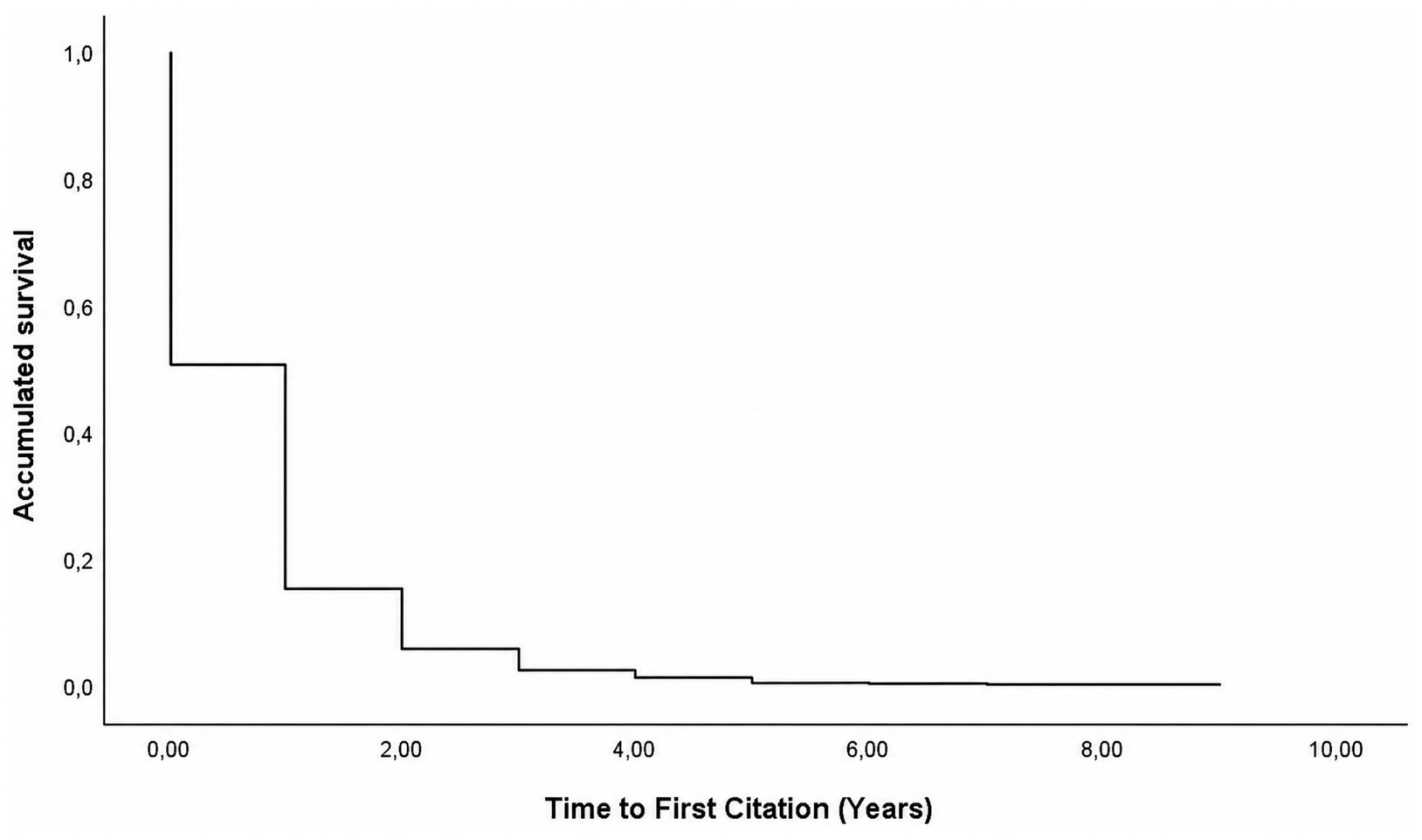
Survival analysis of time to first citation of scientific articles (Kaplan–Meier). This figure illustrates the probability that scientific articles remain uncited over time. The steep decline within the first year indicates that 50% of publications receive their first citation during this period. The curve continues to fall gradually between years 1 and 4, reflecting ongoing citation activity. After year 4, the curve approaches zero, showing that nearly all articles have been cited by this time and that late citations are uncommon.

Figure 4 presents the Kaplan–Meier curve for time to first citation, showing the probability that publications remain uncited over time. The curve declines steeply during the first year, reaching approximately 0.5, indicating that around half of all articles received their first citation within 12 months. It then decreases more gradually between years 1 and 4, before flattening near zero after year 4. Overall, most publications achieved their first citation within 1 to 2 years, whereas articles uncited after 4 years were unlikely to be cited later (Figure 4).

Table 2 presents the results of the univariable and multivariable Cox proportional hazards regression analyses for time to first citation. Of the 746 publications included in the complete-case Cox regression analysis, 607 experienced the event of interest (first citation) during follow-up, whereas 139 remained uncited and were treated as right-censored observations. A total of 3,222 records were excluded from the regression analyses because of missing covariate data (*n* = 3,204) or invalid time values (*n* = 18). The Schoenfeld residual analysis showed no significant violation of the proportional hazards assumption in the overall Cox model (global test: χ^2^ = 5.97, *p* = 0.22). Most variables, including collaboration, open access, article type, and number of authors, demonstrated stable effects over time. Although language showed a slight time-dependent tendency (*p* = 0.04), the overall model remained appropriate for Cox regression analysis (Supplementary Table S1). In the univariable analysis, international collaboration (*HR* = 0.53; 95% *CI*: 0.47–0.59), non-English language (*HR* = 0.38; 95% *CI*: 0.35–0.42), and open access status (*HR* = 0.83; 95% *CI*: 0.77–0.90) were associated with a lower hazard of receiving a first citation, indicating a longer time to first citation. In contrast, original articles (*HR* = 1.34; 95% *CI*: 1.22–1.42) and a higher number of authors (*HR* = 1.001; 95% *CI*: 1.001–1.002) were associated with an increased hazard of receiving a first citation. However, the effect size associated with the number of authors was minimal despite reaching statistical significance.

**Table 2:** Univariable and multivariable Cox proportional hazards regression for time to first citation.

	Univariate analysis (unadjusted *HR*)			Multivariate analysis (adjusted *HR*)		
Variables	Hazard ratio (*HR*)	95% *CI*	*p*-value	Hazard ratio (*HR*)	95% *CI*	*p*-value
Collaborations (yes vs. no)	0.53	0.47–0.59	< 0.001	0.81	0.67–0.97	0.02
Language (Non-English vs. English)	0.38	0.35–0.42	< 0.001	0.53	0.42–0.65	< 0.001
Open access (yes vs. no)	0.83	0.77–0.90	< 0.001	1.11	0.92–1.34	0.26
Article type (original vs. review)	1.34	1.22–1.42	< 0.001	1.40	1.09–1.79	0.007
Number of authors	1.001	1.001–1.002	< 0.001	1.001	1.001–1.002	< 0.001

In the multivariable model, international collaboration remained significantly associated with a lower hazard of receiving a first citation (*HR* = 0.81; 95% *CI*: 0.67–0.97; *p* = 0.02), as did non-English language (*HR* = 0.53; 95% *CI*: 0.42–0.65; *p* < 0.001), indicating longer time to first citation after adjustment. Open access status was no longer significantly associated with citation timing (*HR* = 1.11; 95% *CI*: 0.92–1.34; *p* = 0.26). Conversely, original articles (*HR* = 1.40; 95% *CI*: 1.09–1.79; *p* = 0.007) and a higher number of authors (*HR* = 1.001; 95% *CI*: 1.001–1.002; *p* < 0.001) were associated with a statistically significant but very small increase in the hazard of receiving a first citation, corresponding to a shorter time to first citation.

## 4 Discussion

Citations are widely used in scientometric research as indicators of scholarly impact and visibility (Calò, 2022). Beyond cumulative citation counts, increasing attention has focused on citation dynamics, particularly time to first citation, which captures how quickly newly published research is integrated into academic discourse (Srisawad and Lertsittiphan, 2022). In this study, we applied survival analysis methods to examine early citation patterns in Ecuadorian medical publications, providing empirical evidence from an underrepresented research context.

Time to first citation reflects early research uptake and has been proposed as a sensitive indicator of initial visibility and relevance (Kumari et al., 2020). In our analysis, approximately half of the publications received their first citation within the first year, and most were cited within 4 years. These findings suggest rapid early citation uptake compared to patterns described in some emerging research systems, where longer citation delays have been reported. However, these results should be interpreted cautiously given differences in database coverage, indexing practices, and follow-up time across studies.

The observed citation trajectories are consistent with broader theories of citation delay. Prior work has shown that not all scientific contributions follow linear citation patterns. He et al. (2018) described heterogeneous trajectories, including “second-act papers” and “sleeping beauties,” highlighting that delayed recognition can arise from both intrinsic characteristics of the research and extrinsic contextual factors. Similarly, Zhao et al. (2021), through large-scale co-citation analyses, demonstrated that delayed recognition may emerge from novel combinations of previously disconnected ideas, reinforcing the notion that citation timing reflects more than scientific quality alone.

In the adjusted analysis, international collaboration and non-English language were associated with a lower hazard of receiving a first citation, suggesting a longer time to first citation. In contrast, original articles and a higher number of authors were associated with a slightly increased hazard of receiving an early first citation. Open access status was not independently associated with citation timing after adjustment. Although previous studies have suggested that open access publication may enhance research visibility and citation performance, this association was not observed in the present study. Similarly, while international collaboration has often been linked to greater scientific visibility and dissemination, our findings suggest that internationally collaborative publications experienced a longer time to first citation after controlling for other publication characteristics. These findings differ from previous studies reporting that international collaboration and broader dissemination networks are associated with greater overall citation impact and research visibility (Kumari et al., 2020; He et al., 2021; Zhang et al., 2024). However, citation impact and time to first citation represent distinct dimensions of scientific recognition. Although internationally collaborative publications may accumulate higher citation counts over time, our results suggest that they experienced a longer delay before receiving the first citation.

The progressive increase in cumulative citation counts over time suggests sustained visibility of Ecuadorian medical publications after dissemination. Additionally, the high proportion of internationally collaborative studies may reflect increasing integration of Ecuadorian medical research into global scientific communication networks. Funding has also been suggested as a factor influencing citation recognition, as funded studies may benefit from greater visibility and dissemination resources (Morisawa et al., 2022). However, funding data was not available in our dataset, and this factor could not be directly assessed in the present study.

Despite these patterns, citation speed is not evenly distributed across global research systems. Publications from low- and middle-income countries often experience structural barriers that may delay citation, including limited visibility, publication in lower-impact journals, and language constraints (Crespo et al., 2014). Although previous studies have suggested that open access publication may enhance research visibility (Riera and Aibar, 2013), open access status was not independently associated with citation timing in our adjusted analyses. In this context, Ecuador represents an informative case. Although its medical research system is still developing, the predominance of English-language publications and original research articles may contribute to relatively rapid early citation uptake. However, internationally collaborative publications showed a longer time to first citation after adjustment, suggesting that factors associated with collaboration patterns may influence early citation dynamics differently from overall citation accumulation. These findings are consistent with evidence from other emerging research systems, where alignment with global publishing practices has been associated with improved citation performance (Didegah and Thelwall, 2013).

Methodologically, this study highlights the applicability of survival analysis techniques in scientometric research. The Kaplan–Meier estimator allowed characterization of citation timing, while Cox proportional hazards models were used to explore associations between publication characteristics and time to first citation.

Given the observational design and the limitations in the available metadata, these associations should be interpreted as exploratory rather than causal. Overall, this study provides empirical insight into early citation dynamics in Ecuadorian medical research. While the findings suggest relatively rapid initial citation uptake, they also underscore the importance of structural and publication-related factors in shaping research visibility. Future studies incorporating additional variables, such as funding, institutional characteristics, and alternative metrics, may provide a more comprehensive understanding of citation dynamics in emerging research systems.

## 5 Limitations

This study has several limitations that should be considered when interpreting the findings. First, the analysis was restricted to publications indexed in the Web of Science Core Collection, which may underrepresent articles published in regional or local journals not covered by this database. As a result, citation dynamics of Ecuadorian medical research published outside major indexing platforms may not be fully captured. Second, citation-based indicators reflect academic visibility rather than societal or clinical impact and therefore do not encompass other dimensions of research influence such as policy adoption or changes in clinical practice.

Third, articles published closer to the end of the observation period may have had limited time to accumulate citations, potentially leading to right-censoring despite the use of survival analysis methods. Fourth, some key variables, including open access status, international collaboration, and article type, were not consistently available in the Web of Science metadata. Although a manual verification process was conducted using original publication sources, when possible, incomplete or inconsistent reporting across databases may have introduced classification uncertainty and potential misclassification bias. The complete-case approach resulted in the exclusion of a substantial proportion of records because of missing metadata, which may have reduced statistical power and introduced selection bias.

Fifth, Journal quartile information from Journal Citation Reports (JCR) associated with the Web of Science Core Collection was not available in the exported metadata. Manual retrieval of JCR quartile classifications for all included publications was not feasible due to the large number of articles analyzed. Consequently, journal quartile was not included in the final analyses. Finally, although time to first citation provides valuable insights into early research visibility, it does not capture long-term citation trajectories or late-recognition phenomena, which warrant further longitudinal investigation.

## Data Availability

The raw data supporting the conclusions of this article will be made available by the authors, without undue reservation.
