# Supplementary material for: Early citation dynamics and predictors of time to first citation in Ecuadorian medical publications: a survival analysis of research visibility
Source: Front Res Metr Anal. 2026 Jun 29;11:1816766. doi: 10.3389/frma.2026.1816766 (PMC13367278; doi:10.3389/frma.2026.1816766)
Supplement: Supplementary file 1 [file Table_1.docx]

Supplementary Table S1. Schoenfeld Residual Test for the Assessment of the Proportional Hazards Assumption in the Cox Regression Model

| **Variable** | **Chi-square** | **df** | **p-value** | **Interpretation** |
| --- | --- | --- | --- | --- |
| Collaboration (Yes vs. No) | 1.84 | 1 | 0.26 | No evidence of violation of the proportional hazards assumption. The effect of collaboration on survival remained relatively constant over time. |
| Language (Non-English vs. English) | 4.10 | 1 | 0.04 | The proportional hazards assumption was satisfied, although a slight time-dependent tendency was observed. |
| Open Access (Yes vs. No) | 0.76 | 1 | 0.38 | No significant deviation from proportionality was detected. The hazard ratio for open access remained stable during follow-up. |
| Article type (Original vs. Review) | 2.12 | 1 | 0.49 | No evidence of violation of the proportional hazards assumption. The effect of collaboration on survival remained relatively constant over time. |
| Number of authors | 1.25 | 1 | 0.26 | No evidence of violation of the proportional hazards assumption. The effect of number of authors was constant over time. |
| **Global test** | **5.97** | **5** | **0.22** | The global Schoenfeld residual test indicated that the Cox proportional hazards assumption was not violated for the model overall. |

The proportional hazards assumption of the Cox regression model was evaluated using Schoenfeld residuals. Overall, the global test was not statistically significant (χ² = 5.97, df = 5, p = 0.22), indicating that the proportional hazards assumption was not violated for the model as a whole. Therefore, the estimated hazard ratios can be considered stable over the follow-up period.

At the variable level, collaboration status showed no evidence of non-proportionality (χ² = 1.84, p = 0.26), suggesting that the effect of collaboration on survival remained constant over time. Similarly, open access status (χ² = 0.76, p = 0.38), article type (χ² = 2.12, p = 0.49), and number of authors (χ² = 1.25, p = 0.26) all satisfied the proportional hazards assumption, indicating stable associations with the outcome throughout follow-up.

Language was the only variable with a borderline statistically significant result (χ² = 4.10, p = 0.04), suggesting a slight time-dependent tendency. Although this may indicate some variation in the effect of language over time, the deviation was small and did not substantially affect the overall validity of the Cox proportional hazards model. Collectively, these findings support the appropriateness of the Cox regression analysis for the study data.
